# Subtotal glossectomy with conservation of the hyo-styloglossus unit (HSU): a new pivotal concept for preserving tongue function in extended glossectomy

**DOI:** 10.3389/fsurg.2024.1395936

**Published:** 2024-07-04

**Authors:** Luca Gazzini, Arianna Caselli, Virginia Dallari, Enrico Fazio, Monir Abousiam, Aurel Nebiaj, Cecilia Albi, Remo Accorona, Armando De Virgilio, Antonio Greco, Luca Calabrese

**Affiliations:** ^1^ Department of Otorhinolaryngology-Head and Neck Surgery, Hospital of Bolzano (SABES-ASDAA), Teaching Hospital of Paracelsus Medical University (PMU), Bolzano-Bozen, Italy; ^2^ Speech and Language Therapy Division, Department of Otorhinolaryngology-Head and Neck Surgery, Hospital of Bolzano (SABES-ASDAA), Teaching Hospital of Paracelsus Medical University (PMU), Bolzano-Bozen, Italy; ^3^ Unit of Otorhinolaryngology, Head & Neck Department, University of Verona, Verona, Italy; ^4^ Division of Otorhinolaryngology-Head and Neck Surgery, University of Ferrara, Ferrara, Italy; ^5^ Unit of Otorhinolaryngology, ASST Grande Ospedale Metropolitano Niguarda, Milano, Italy; ^6^ Department of “Organi di Senso”, University “Sapienza”, Rome, Italy

**Keywords:** compartmental surgery, oral tongue cancer, tongue surgery, anatomic-based surgery, reconstructive surgery

## Abstract

**Objective:**

The local spread of oral tongue squamous cell carcinoma (OTSCC) follows pathways of dissemination along areas of lesser resistance. In more advanced scenarios, the tumor can extend beyond the hemi-tongue of origin, by passing through the lingual septum and following the fibers of the transverse muscle. This can lead to the invasion of the contralateral extrinsic muscles, the first being the genioglossus and more laterally the hyoglossus. An anatomically guided surgical resection of the tumor can be planned to ensure both oncological safety and an acceptable functional outcome. This approach aims to preserve the hyo-styloglossus unit (HSU) whenever feasible.

**Methods:**

Between January 2019 and November 2022, six patients received extended glossectomy Type B (EG Type B), with preservation of the HSU. Preliminary oncological results and functional results in terms of swallowing (FOIS score) and quality of life (MDADI) are presented.

**Results:**

Five out of the six patients are alive and disease-free, while one patient died due to other causes. All patients who were candidates for an EG Type B underwent a swallowing assessment prior to surgery and followed daily postoperative swallowing training. At discharge, the patients continued swallowing training in an outpatient clinic. Five out of the six patients reached a full oral diet within 1 year of follow-up.

**Conclusion:**

The oncological results confirm the safety of this technique. The importance of preserving the HSU, the minimal functional unit, shows very encouraging results in terms of swallowing rehabilitation.

## Introduction

Advanced oral tongue squamous cell carcinoma (OTSCC) represents a challenge for head and neck surgeons. The goal of surgery is to achieve oncological radicality while ensuring acceptable functional results in terms of speech and swallowing. In advanced tumors (cT3-T4a), the disease can spread through the lingual septum and along the intrinsic transverse muscle to involve the contralateral hemi-tongue (1). An anatomic-based approach, performed according to preoperative imaging and intraoperative findings, allows for complete and oncologically safe resection, preserving structures not involved in the disease.

**Figure 1:**
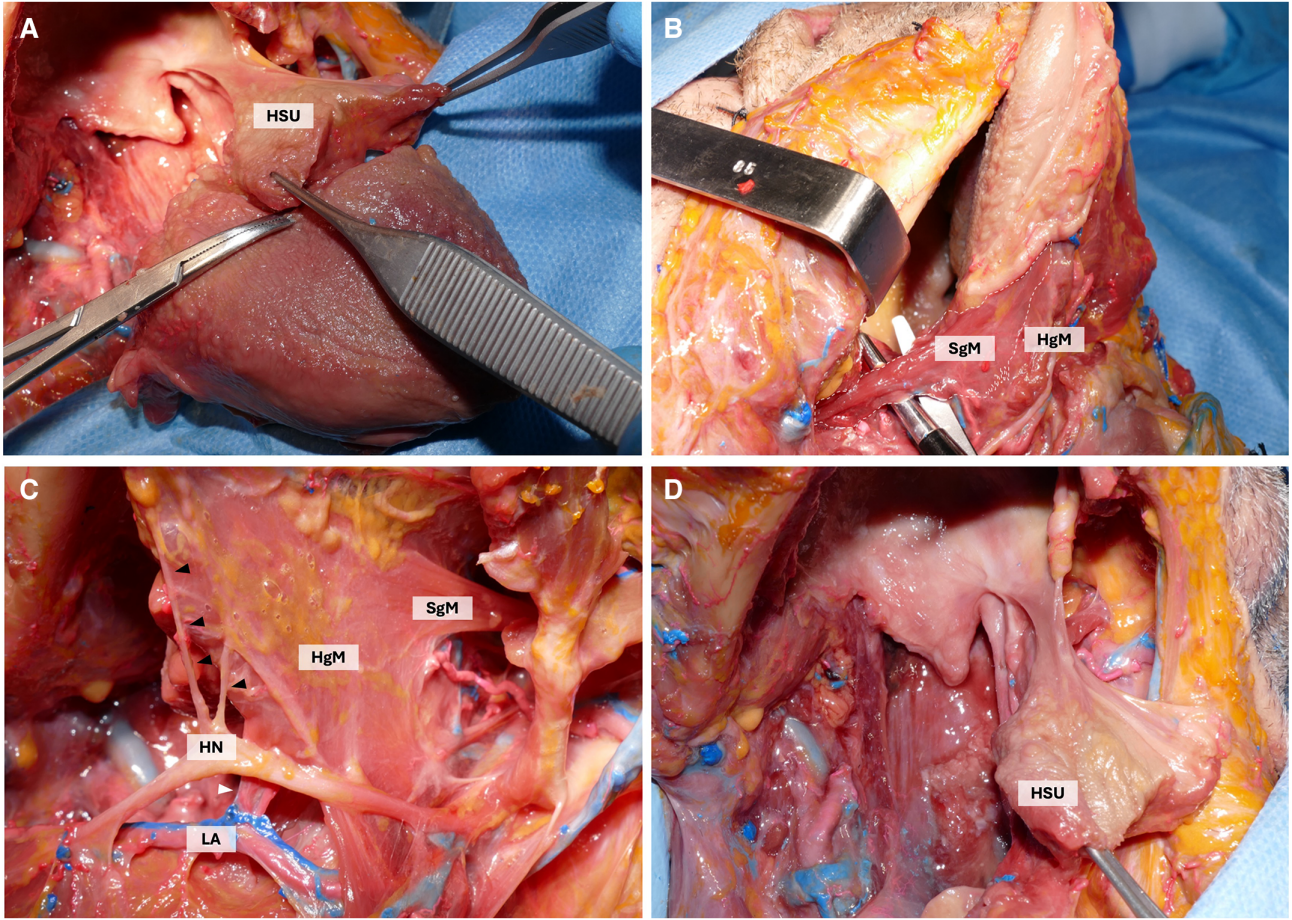
Cadaveric dissection highlighting the HSU. (**A**) Isolation of the intrinsic musculature of the HSU. (**B**) lateral view highlighting the course of the styloglossus muscle (SgM) whose fibers merge with those of the hyoglossus muscle (HgM). (**C**) Lateral view of the HSU highlighting the branches of the hypoglossal nerve (HN) (black arrowhead) and of the lingual artery (LA) (white arrowhead) supplying the HSU. Styloglossus muscle (SgM). (**D**) the HSU after the subtotal glossectomy.

In advanced OTSCC, one of the main adverse features in predicting postoperative swallowing impairment is the involvement of the base of the tongue (BOT). The key role of the BOT in maintaining an acceptable swallowing function was already demonstrated in the literature by the experience of Chen et al. in robotic surgery (2, 3). We postulated that to maintain an acceptable swallowing function, it is paramount to preserve a functional unit made by the hyoglossus and styloglossus muscles together with their innervation, for which the term hyo-styloglossus unit (HSU) was coined (Figure 1).

The study aims to describe the surgical approach to advanced cT4a tumors that extend beyond the lingual septum to the contralateral hemi-tongue involving the genioglossus muscle while sparing the hyoglossus and styloglossus muscle of the contralateral side. The procedure of the subtotal glossectomy sparing the HSU (EG Type B) is described step by step, focusing on the surgical anatomy. In addition, preliminary functional results in terms of swallowing and quality of life (QoL) are presented.

## Materials and methods

A retrospective review of patients who underwent EG Type B for advanced OTSCC between January 2019 and November 2022 was conducted in the Department of Otolaryngology-Head and Neck Surgery in “Ospedale Centrale” of Bolzano (4).

All patients underwent preoperative MRI with gadolinium, which is a fundamental decision-making tool to depict the invasion of key anatomical structures.

**Figure 2:**
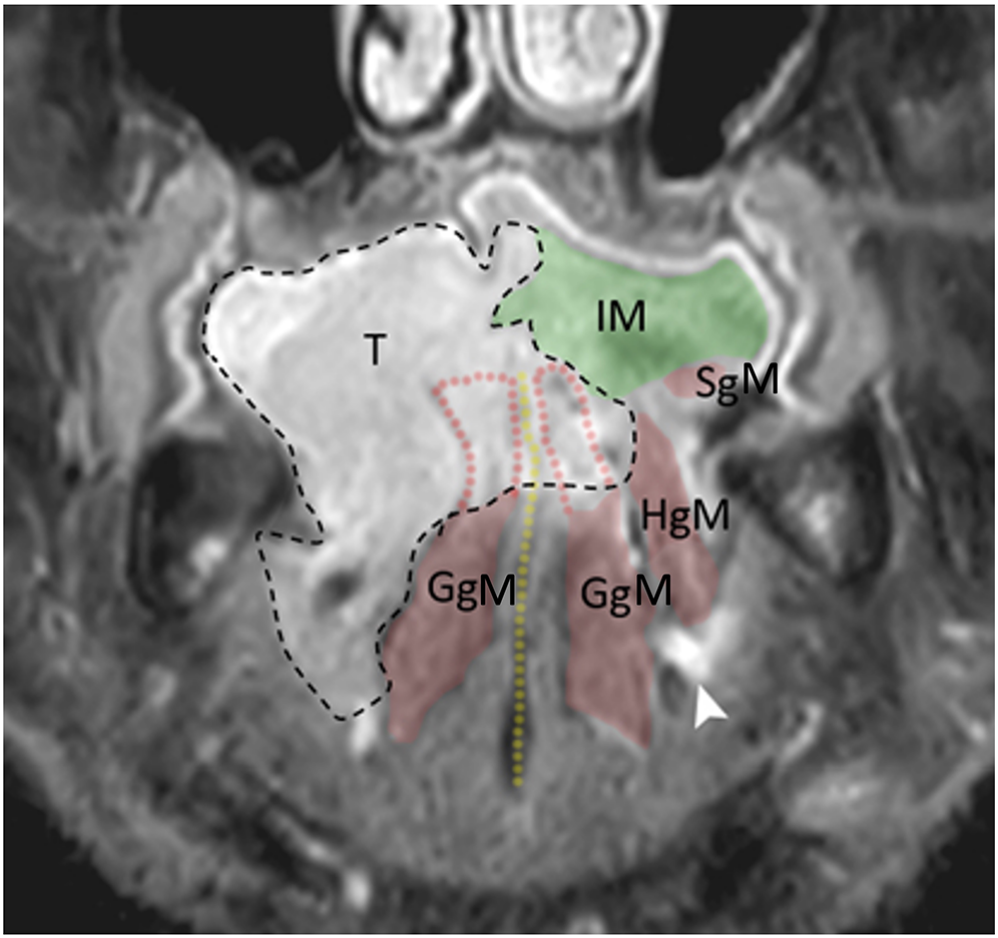
MRI sagittal view: tumor enveloping the genioglossus muscle on both sides. T, tumor; IM, intrinsic musculature; SgM, styloglossus muscle; GgM, genioglossus muscle; HgM, hyoglossus muscle. Yellow dotted line, lingual septum; black dotted line, tumor border.

Based on the clinical presentation and MRI, the indications for subtotal glossectomy with preservation of the HSU are as follows:

○ advanced cT3-cT4a OTSCC extended beyond the lingual septum and involving the contralateral hemi-tongue (Figure 2);
○ bilateral involvement of the genioglossus muscle, with sparing of the contralateral hyoglossus, styloglossus muscle, and hypoglossal nerve; and
○ general patient conditions permitting major surgery and reconstruction with a microvascular free flap.

### Surgical technique

**Figure 3:**
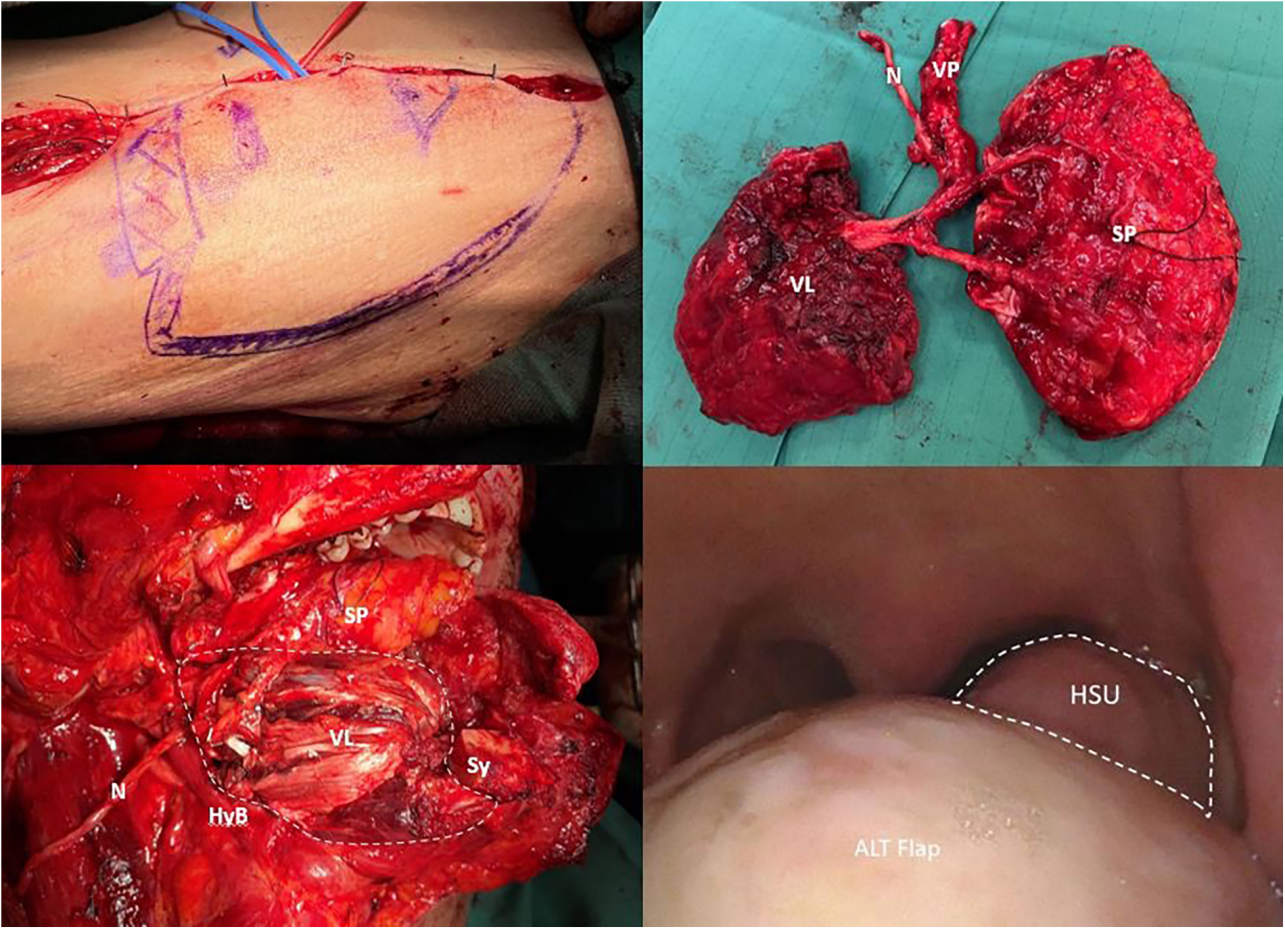
Insetting of the ALTF. The white dotted line indicates the preserved HSU. N, nerve; VP, vascular pedicle; VL, vastus lateralis muscle; SP, skin paddle; HyB, hyoid bone; Sy, mental symphysis.

An ipsilateral CTS is performed, following the surgical steps of the standard technique already described in the literature (5, 6). The only difference is that in this case the dissection of the lingual septum must be avoided. At this point, the macroscopic invasion of the contralateral genioglossus muscle can be assessed under direct vision. We describe thereby the surgical approach to the contralateral compartment of the tongue (7):

○ The first step is the detachment of the insertion of the contralateral genioglossus muscle from the mental symphysis.
○ The contralateral lingual artery is identified by using the genioglossus muscle surface as the dissection plane to locate it. The lingual artery runs between the lateral aspect of the genioglossus muscle and the medial aspect of the hyoglossus and styloglossus muscles.
○ Taking the lingual artery as a landmark, the hyoglossus and styloglossus muscles are identified superficially in relation to the vascular space. The hyoglossus muscle is located in an anterior position, while the styloglossus muscle is located posteriorly and its distal fibers merge anteriorly with the lateral fibers of the hyoglossus muscle. These two muscle structures, together with the terminal branches of the hypoglossal nerve directed to them, represent the HSU. During the surgical dissection, care has to be taken to preserve the HSU in order to maintain its anatomical integrity and thus the functionality of the BOT.
○ The sublingual region is dissected, identifying the sublingual gland, which is cleared of surrounding stromal tissue. Moreover, lingual and hypoglossal nerves are identified and their terminal branches directed to the genioglossus muscle are sectioned. Care must necessarily be taken to preserve the branches for the hyoglossal and styloglossal muscles in order to maintain motor innervation to the HSU.
○ The last step is the detachment of the genioglossus from the hyoid bone. Finally, the resection of the dorsal aspect of the tongue with the mucosa and intrinsic musculature is performed with a monopolar scalpel.
○ The reconstruction is conducted using a microvascular free flap. Our workhorse flap is the anterolateral thigh flap (ALTF), which gives the advantage of harvesting a chimeric flap with the vastus lateralis muscle. This is particularly important in these patients in order to supply enough bulk of the tongue base, to achieve a proper swallowing during the pharyngeal phase (Figure 3). The suspension of the larynx, a fundamental step in swallowing recovery, is ensured by the contralateral digastric and stylohyoid muscles, which support the hyoid bone. On the ipsilateral side, the inset of the flap is performed as follows: the vastus lateralis muscle is fixed between the hyoid bone and the mandibular symphysis to achieve both flap support and suspension of the central part of the hyoid bone to the mandible. The fascia lata harvested with the flap is instead fixed between the lateral portion of the hyoid body and the mandible body, ensuring laryngeal suspension. In case the contralateral digastric muscle is also sectioned, hyoid suspension via the flap's fascia lata is made contralaterally as well.

### Rehabilitation protocol and evaluation of swallowing and QoL

All patients eligible for an EG Type B underwent swallowing assessment before surgery and received daily postoperative swallowing training with a speech and language therapist (SLT). At discharge, the patients continued swallowing training in an outpatient clinic. The goal of the swallowing rehabilitation is to obtain a functional swallowing act, which allows patients to reach oral feeding safety and the absence of aspiration pneumonia. The patients learn with an SLT how to manage oral and pharyngeal residue.

The parameters measured in this research were functional oral intake ability and the patient's QoL. Swallowing function was assessed four times: pre-surgery, after 2 weeks of rehabilitation, 6 months post-surgery, and during long-term follow-up (at least 1 year post-surgery), using the Italian version of the Functional Oral Intake Scale (FOIS) (8). To assess the impact of oral dysphagia on quality of life, an Italian version of the M.D. Anderson Dysphagia Inventory (MDADI) by Chen et al. was used (9). The MDADI was self-administered at least 1 year post-surgery in a quiet room and in the presence of the SLT in case the patient required clarifications.

## Results

Six patients (five males and one female, with a median age of 49.2 years) were included in the study. Two patients were active smokers, whereas none had a history of alcohol abuse. All patients were affected by advanced tongue squamous cell carcinoma pT3 or pT4a. Data about patients, surgery, and follow-up are summarized in Table 1. The types of consistencies of foods and liquids are categorized according to the International Dysphagia Diet Standardisation Initiative (IDDSI) Framework of 2016 (10).

**Table 1:** Patient, tumor, and surgical data.

	Age	Gender	Site	Clinical stage	Comorbidities	Risk factors	Surgery	Neck dissection	Reconstruction	Histology	Pathological classification
Patient 1	70 years	Male	Right floor of the mouth	cT4aN2cM0	Chronic kidney disease and obesity (BMI 32)	No history of tobacco or alcohol abuse	Extended glossectomy Type B with segmentary mandibulectomy	Bilateral MRND I–V	Chimeric left ALTF with vastus lateralis muscle	SCC G3 p16+	pT4a N3b R0 V1 L1 pn1
Patient 2	35 years	Male	Right tongue margin	cT3N2bM0	None	No history of tobacco or alcohol abuse	Transmandibular extended glossectomy Type B	Bilateral MRND levels I–V	ALTF	SCC G2 p16-	pT4a N2c R0 V1 L1 pn1
Patient 3	26 years	Male	Right tongue, OTSCC G2 pT2N0R0, that underwent surgery and adjuvant RT in another center	Relapse in the TN tract cT3N0M0	None	No history of tobacco or alcohol abuse	Pull through extended glossectomy Type B	Previous right SND I–III	Chimeric left ALTF with vastus lateralis muscle	SCC G3	pT3 R0 V0 L0
Patient 4	39 years	Male	Left tongue and oropharynx left	cT3N0M0	None	Smoker, no alcohol abuse	Transmandibular extended glossectomy Type B with extension to the oropharynx left	Left SND IIA–IV	Chimeric left ALTF with vastus lateralis muscle	SCC G2 p16-	pT3 N0 R0 V0 L0
Patient 5	83 years	Male	Right tongue and oropharynx	cT3N0M0	Severe Neurocognitive disorder, Bladder neoplasm	Smoker, no alcohol abuse	Pull through extended glossectomy Type B with extension to the oropharynx right	Right MRND I–V	Major Pectoralis Flap	SCC G3 p16-	pT3 N0 R0 V0 L0
Patient 6	42 years	Female	Left tongue, that underwent exclusive RT in another center.	cT3N3M0	None	Non-smoker, no alcohol abuse	Pull through extended glossectomy Type B with preservation of HSU on both sides	Right RND I–V and left SND I–IV	Chimeric left ALTF with vastus lateralis muscle	SCC G3 p16-	pT3 N3b R0 V1 L1

	Postoperative complications	Tracheostomy tube removal	Start of swallowing training	NGT removal	Hospital discharge	Oral intake at discharge	Need for peg	Adjuvant therapies	Long-term oral intake	Last follow-up	Oncological status
Patient 1	None	7th postoperative day	7th postoperative day	8th postoperative day	35th postoperative day	Small quantities of mildly thick foods and drunk small quantities of thin liquids, the oral and pharyngeal residue was removed by drinking thin liquids	Yes, during and post adjuvant RT	Adjuvant RT. CHT was not indicated for chronic kidney disease	Five months post-surgery pureed foods (IDDSI 4) and six months post-surgery minced and moist foods (IDDSI 5), thin liquids (IDDSI 0). Full oral intake after 1 year and removal of PEG	25 months	Disease-free, no history of development of aspiration pneumonia
Patient 2	None	6th postoperative day	8th postoperative day	13th postoperative day	17th postoperative day	Completely eating a pureed diet by mouth and drinking thin liquids. Took oral nutritional supplements. Pharyngeal residue was removed by drinking thin liquids.	No	Adjuvant RT and CHT	At one year follow-up the patient was eating a full oral diet (IDDSI 6) and drinking thin liquids (IDDSI 0)	46 months	Disease-free, no history of development of aspiration pneumonia
Patient 3	Sinusal tachycardia. Resolved with beta-block.	7th postoperative day	7th postoperative day	25th postoperative day	15th postoperative day	Small quantities of pureed foods and thin liquids by mouth. Pharyngeal residue was removed by drinking thin liquids	No	None (previous RT)	At one year follow-up the patient was eating a full oral diet (IDDSI 6) and drinking thin liquids (IDDSI 0)	13 months	Relapse at pre laryngeal cutaneous level, surgical resection. Disease-free, no history of development of aspiration pneumonia
Patient 4	None	8th postoperative day	6th postoperative day	15th postoperative day	20th postoperative day	Completely eating a pureed diet by mouth and drinking thin liquids. Took oral nutritional supplements. Pharyngeal residue was removed by drinking thin liquids	No	Adjuvant RT	At one year follow-up the patient was eating a full oral diet (IDDSI 6) and drinking thin liquids (IDDSI 0)	38 months	Disease-free, no history of development of aspiration pneumonia
Patient 5	Macroematuria, urosepsis (E.Coli) treated with antibiotic therapy.	11th postoperative day	15th postoperative day	24th postoperative day, position of PEG	38th postoperative day	Small quantities of mildly thick foods and drunk small quantities of thin liquids with the supervision of SLT/caregiver	Yes	None	Small quantities of mildly thick foods and drunk small quantities of thin liquids with supervision of caregiver	42 months	Deceased of other causes (urosepsis)
Patient 6	Wound infection treated conservatively with antibiotics	10th postoperative day	7th postoperative day	14th postoperative day	20th postoperative day	Completely eating mildly thick foods by mouth and drinking thin liquids. Took oral nutritional supplements. Pharyngeal residue was removed by drinking thin liquids	No	No	At 6 months follow-up the patient was mildly thick foods by mouth (IDDSI 4) and drinking thin liquids (IDDSI 0)	6 months	Disease-free, no history of development of aspiration pneumonia

ALTF, anterolateral thigh flap; RT, radiotherapy; CHT, chemotherapy; SCC, squamous cell carcinoma; MRND, modified radical neck dissection; SND, selective neck dissection; NGT, nasogastric tube; PEG, percutaneous endoscopic gastrostomy.

Regarding the swallowing results, five out of the six patients reached a full oral diet within 1-year follow-up, with a score of 6 on the FOIS. One patient with severe neurocognitive disorder continued to be fed through a percutaneous gastrostomy, since sufficient oral intake was not reached. Information regarding swallowing function and the patient's QoL is summarized in Tables 2, 3.

**Table 2:** Evaluation of swallowing outcomes (FOIS score).

	Patient 1	Patient 2	Patient 3	Patient 4	Patient 5	Patient 6
	FOIS	FOIS	FOIS	FOIS	FOIS	FOIS
Pre-surgery	6	7	6	7	5	6
Post-surgery (after 2 weeks of rehabilitation)	2	5	3	5	2	5
After 6 months post-surgery	3	5	5	5	2	5
Long-term (at least 1 year post-surgery)	6	6	6	6	2	6

6 = Total oral intake with no special preparation, but must avoid specific food or liquid items = patients avoid solid and dry foods.

5 = Total oral intake of multiple consistencies requiring special preparation = patients need to take small pieces of soft foods.

**Table 3:** QoL outcome scores of the MDADI.

	Patient 1	Patient 2	Patient 3	Patient 4	Patient 5	Patient 6
Global score	4/5	5/5	4/5	4/5	^a^	4/5^b^
Composite score	67/100	95/100	85/100	90/100	^a^	80/100^b^

^a^ Administration not possible (impaired cognitive status).

^b^ Administration 6 months post-surgery.

## Discussion

The primary goal of tumor ablation in OTSCC is to remove the entire tumor along with a “margin” of normal tissue. Inadequate clearance of tumor cells results in increased risks of local and regional recurrences, as well as decreased long-term survival rates (11, 12).

The extrinsic muscles of the tongue can be divided into a peripheral group including palatoglossus, styloglossus, and hyoglossus muscles, which are located laterally, and the genioglossus muscle, located in a deeper plane, which gives rise to fibers running to the periphery, the dorsal surface, and the apex of the tongue (13). Local tumor spread first involves the intrinsic musculature following the direction of muscle fibers, the genioglossus muscle in case of an anterior tumor, or the hyoglossus and styloglossus muscles in case of a posterior tumor. In the case of macroscopic infiltration of the extrinsic musculature of the tongue, CTS is indicated, with the aim of removing the entire oncological compartment with the pathways of tumor spread. These include the tumor, within the entire hemi-tongue and related floor of the mouth and the neck lymph nodes in continuity with the so-called tumor-node tract (TNT) (5, 12).

The lingual septum represents a barrier to contralateral spread, but it is not uncommon in advanced stages (cT3-cT4a) for it to be overcome. In those cases, the extension of the resection to the contralateral tongue may affect the postoperative tongue functionality and thus the patient's quality of life. The defect resulting from EG Type B requires reconstruction that includes free flaps to adequately restore bulk and prevent ankyloglossia (13). Radical surgical treatment often affects all oral functions, such as speech and swallowing physiology (13, 14). In the oral preparatory phase, there can be alterations related to tongue motility for bolus preparation and formation and, in the oral phase, alterations related to bolus transportation and bolus control. In the pharyngeal phase, alteration of swallow initiation, posterior tongue propulsion, hyolaryngeal excursion, bolus propulsion, post-swallow pharyngeal residue, and risk of penetration and aspiration can be observed (15, 16). Advanced tumor (T) stage, extraoral surgical approach, the incremental volume of glossectomy defect, and adjuvant radiotherapy correlate significantly with poor swallowing outcomes (17, 18).

Grammatica et al. investigated the long-term functional outcomes in patients treated by CTS and reconstructed by free flaps and demonstrated that CTS does not significantly affect speech and swallowing (5). However, the study included only patients who underwent ipsilateral CTS.

The important role of the BOT in the propulsion of food makes its removal of particular concern, even if tongue base resections are becoming increasingly common in the modern era of transoral robotic surgical approaches (2, 3).

In this scenario, the pivotal concept of HSU in the maintenance of an acceptable residual swallowing function should be highlighted. The HSU can be compared to the cricoarytenoid unit in partial OPHL laryngectomies, where preservation of one arytenoid with the underlying portion of the cricoid plate and the recurrent nerve allows normal motility of the arytenoid (functioning cricoarytenoid unit) (19, 20). A concept similar to HSU has been described in the works of Gawryszuk et al. in the field of radiotherapy (21, 22). They introduced a concept rooted in anatomy and physiology known as functional swallowing units (FSU), aiming to enhance comprehension of radiation-induced dysphagia.

Preserving HSU is one of the main goals of the EG Type B also in the case of bilateral involvement of the genioglossus muscles. This unit must only be sacrificed if there is a direct invasion of the hyoglossus muscle bilaterally, so in this case, the approach should be converted to total glossectomy.

The importance of preserving the HSU in advanced tongue tumors is supported by our encouraging results in terms of swallowing and QoL. Though the number of patients is limited, it is noteworthy that all patients but one resumed a regular oral food intake of liquid and solid of different consistencies, and did not develop aspiration pneumonia. All patients, except one, can swallow liquids and foods of different consistencies using the swallowing technics learned during rehabilitation. The long-term results regarding the impact of dysphagia on their QoL demonstrated that all patients present good day-to-day functioning and QoL.

In our series, only one patient with severe comorbidities was not reconstructed with a free microvascular flap but in a sub-optimal way with a major pectoralis flap. Furthermore, he was affected by a severe cognitive disorder that prevented swallowing rehabilitation. This was the only patient in our case series that was not able to resume a full oral diet and a percutaneous endoscopic gastrostomy was performed and maintained. This experience confirms that patients who are candidates for surgery should be carefully selected, including only those who are suitable for a highly demolitive procedure, microvascular reconstruction, and an intensive swallow rehabilitation program. Therefore, patients should be appropriately counseled on the risks of this strong demolitive surgery with emphasis placed on intensive swallow rehabilitation in the posttreatment setting (2, 13).

Regarding the oncological outcomes, as shown in Table 1, all patients thus far are disease-free except for one who passed away due to other reasons. These results demonstrate that preserving the HSU, which we have observed to be both functional and essential for swallowing, does not compromise oncological safety.

Further studies are needed to confirm the preliminary functional results shown in the present study.

## Conclusion

In our experience, the minimal functional unit that allows an acceptable swallowing result is the so-called functional unit of the BOT, which includes the hyoglossus and styloglossus muscles innervated by the hypoglossal nerve (HSU).

The importance of preserving this functional unit is corroborated by the highly encouraging results we observed in terms of both swallowing and QoL.

## Data Availability

The raw data supporting the conclusions of this article will be made available by the authors, without undue reservation.
